# Mesoporous silica nanoparticle-based intelligent drug delivery system for bienzyme-responsive tumour targeting and controlled release

**DOI:** 10.1098/rsos.170986

**Published:** 2018-01-10

**Authors:** Yang Zhang, Juan Xu

**Affiliations:** Department of Obstetrics and Gynecology, Tengzhou Central People's Hospital, No. 181 Xingtan Road, Shandong 277599, People's Republic of China

**Keywords:** drug delivery, mesoporous silica, tumour targeting, gelatin, hyaluronic acid, bienzyme-responsive

## Abstract

This paper proposes a novel type of multifunctional envelope-type mesoporous silica nanoparticle (MSN) to achieve cancer cell targeting and drug-controlled release. In this system, MSNs were first modified by active targeting moiety hyaluronic acid (HA) for breast cancer cell targeting and hyaluronidases (Hyal)-induced intracellular drug release. Then gelatin, a proteinaceous biopolymer, was grafted onto the MSNs to form a capping layer via glutaraldehyde-mediated cross-linking. To shield against unspecific uptake of cells and prolong circulation time, the nanoparticles were further decorated with poly(ethylene glycol) polymers (PEG) to obtain MSN@HA-gelatin-PEG (MHGP). Doxorubicin (DOX), as a model drug, was loaded into PEMSN to assess the breast cancer cell targeting and drug release behaviours. *In vitro* study revealed that PEG chains protect the targeting ligand and shield against normal cells. After reaching the breast cancer cells, MMP-2 overpressed by cells hydrolyses gelatin layer to deshield PEG and switch on the function of HA. As a result, DOX-loaded MHGP was selectively trapped by cancer cells through HA receptor-mediated endocytosis and subsequently release DOX due to Hyal-catalysed degradation of HA. This system presents successful bienzyme-responsive targeting drug delivery in an optimal fashion and provides potential applications for targeted cancer therapy.

## 1. Introduction

For cancer chemotherapy, one of the major challenges is how to transport medicinally active molecules to their molecular site of action [1–4]. As traditional antineoplastic drugs exhibit poor pharmacokinetic profiles and broad mechanisms of action, they carry a substantial risk of systemic toxicity with undesired side effects [5,6]. Drug delivery systems (DDSs) based on nanotechnology present a great potential to revolutionize cancer chemotherapy [7–11]. Compared with conventional small-molecule antineoplastic drugs, DDSs can protect the drug from premature degradation, prevent drugs from prematurely interacting with the biological environment, and control the pharmacokinetic and drug tissue distribution profile [12,13]. Despite these burgeoning achievements, DDSs also encounter several transport barriers on their tortuous journey to tumoural cells, such as the aggregation during the circulation, rapid clearance via the reticulo-endothelial system (RES) and unspecific uptake of normal cells.

To negotiate these barriers, a practical DDS should possess the two general characteristics of prolonged circulation time and highly specific targeting-uptake of cancer cells. With regard to prolonged circulation time, it is well proved that hydrophilic PEGylation can not only sterically stabilize particles but also provide ‘stealth function’ to DDSs for escape of RES. So, the PEGylated nanoparticles can remain in blood circulation, which is the precondition of drug delivery [14–17]. With regard to highly specific targeting-uptake of cancer cells, bioconjugation of cell-specific ligands to the surface of nanoparticles, such as antibodies [18], peptides [19], aptamers [20,21], small molecules [22], can retain the nanoparticles in tumours and promote endocytic uptake by target cells. Up to now, enormous research efforts have been devoted to designing DDS-combined poly(ethylene glycol) polymers (PEGs) and cell-specific ligands [23,24]. Most of the designs are based on cell-specific ligand grafting at the end of PEGs [25]. But these inflexible designs could not realize the optimal drug delivery because the PEG layer inhibits the interaction of the DDSs with the tumour cell surface, resulting in minimal cellular uptake and significant loss of activity for the delivery system [26]. Therefore, it is a dire need to develop new strategies to maximize the functions of PEG and cell-specific ligands for the enhanced drug delivery efficiency.

Recently, a new strategy called ‘multifunctional envelope-type nanodevice (MEND)’ has been proposed for designing intelligent DDS to overcome the biological barriers in the process of drug delivery [27]. To do this, antineoplastic agents are firstly loaded into nanoparticles. Then, the functional components are grafted on the surface of nanoparticles based on the fundamental understanding of these biological barriers [28]. Targeting ligands are firstly protected by a stealth layer for prolonging the systemic circulation time. When the particles reach the tumour site in systemic circulation, the stealth layer is removed in response to the local environment of tumour. As a result, targeting ligands in the innermost layer are exposed and bind target receptors of cancerous cells, which helps to retain the nanoparticles within the tumour. MEND is a rational strategy for achieving optimum pharmacokinetics and pharmacodynamics because the functional components can fully exert their functions at the right time and at the right site. For example, Zhang *et al.* fabricated a tumour-triggered targeting DDS in programmed packing manner using mesoporous silica nanoparticles (MSNs) as drug carriers because of their robust frameworks, ease of functionalization and good biocompatibility [29]. In this system, β-Cyclodextrin (β-CD) was grafted on MSNs via disulfide linking for glutathione-induced intracellular drug release. Then Arg-Gly-Asp (RGD) motif (targeting ligand), tumour microenvironment-responsive substrate peptide Pro-Leu-Gly-Val-Arg and poly(aspartic acid) protection layer were introduced onto the surface of the nanoparticles for exerting specific functions of these components at appropriate site and time. Zou *et al*. developed an intelligent MSN nanocarrier for matrix metalloprotease 2 (MMP-2)-triggered tumour targeting and release by integrating MEND strategy and MMP-2-degradable gelatin [30]. Nevertheless, there still are some disadvantages in terms of the complexity of synthetic and purification process, unsatisfactory pre-release in an extracellular environment, costliness of raw materials, non-natural origin and immunogenicity of functional units etc. Thence, the development of intelligent DDSs by a MEND strategy remains an exciting challenge.

**Scheme 1.**
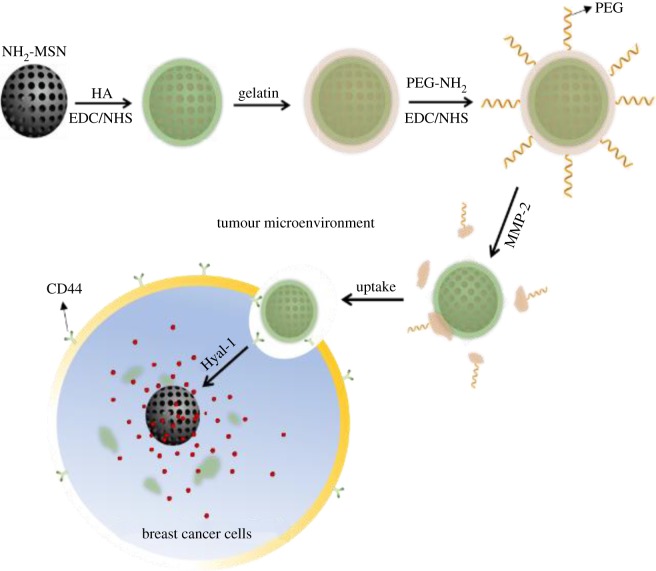
Schematic illustration of the formation of multifunctional drug-loaded MHGP and bienzyme-responsive tumour targeting and controlled release.

Herein, we propose a novel intelligent DDS for microenvironment-responsive tumour targeting and controlled release based on the MEND strategy, MMP-2-triggered degradation of gelatin and hyaluronidases (Hyal)-catalysed degradation of hyaluronic acid (HA). In this system, MSN was selected as a suitable drug carrier. Some functional agents, Hyal-degradable HA capping layer, MMP-2-degradable gelatin and PEG, were packaged onto MSN programmatically to form MSN@HA-gelatin-PEG (MHGP) for achieving bienzyme-responsive targeted drug delivery in an optimal fashion. The structure and delivery strategy of multifunctional MHGP are illustrated in scheme 1. Doxorubicin (DOX) hydrochloride, an anti-cancer drug, was loaded in the mesoporous silica core with the surface modified with HA using 1-ethyl-3-[3-dimethylaminopropyl] carbodiimide hydrochloride (EDC)/sulfo-NHS coupling chemistry. Subsequently, the HA capping onto the DOX-loaded MSN was performed through glutaraldehyde-mediated cross-linking. Finally, the nanoparticle was conjugated with amino-PEG to form MHGP. When MHGP was injected *in vivo*, PEG provide stealth feature to DDS for avoiding the nonspecific uptake. After the nanoparticle arrives at tumour sites, the PEG outlayer can be removed via the MMP-triggered hydrolyzation of the gelatin, leading to the exposure of HA. HA, a biodegradable, biocompatible and non-immunogenic glycosaminoglycan, can work as a targeting moiety for cancer therapy because many types of tumour cells over-express HA receptors like CD44, RHAMM (receptor for hyaluronan-mediated motility, CD168, involved in wound healing and cancer), and HARE (HA receptor for endocytosis). So, HA-modified delivery systems can enter cells more efficiently via the HA receptor-medicated endocytosis pathway. Thereafter, HA was degraded by lysosomal enzyme hyaluronidase, which is a major enzyme found in the tumour microenvironment, into low-molecular-weight fragments after being endocytosed by cancer cells. As a result, the drug could be released from the nanoparticle because of the removal of the HA capping layer. The multifunctional MHGP can exert its specific functions at appropriate sites at the right time, which significantly enhance the efficacy of anti-cancer drug and reduce the cytotoxicity.

## 2. Experimental section

### 2.1. Chemicals and materials

Tetraethylorthosilicate (TEOS, 28%), *N*-Cetyltrimethylammonium bromide (CTAB) and (3-aminopropyl)triethoxysilane (APTES) were purchased from Alfa-Aesar. Gelatin, glutaraldehyde, *N*-hydroxysuccinimide (NHS), *N*-(3-dimethylaminopropyl)-*N*-ethylcarbodiimide hydrochloride (EDC) and 3-[4,5-dimethylthiazol-2-yl]-2,5-diphenyltetrazolium bromide (MTT) were purchased from Sigma-Aldrich. Doxorubicin (DOX) hydrochloride , sodium hyaluronate (HA) (MW = 200 kDa) was purchased from Aladdin. Heochst-33342 was purchased from Invitrogen Life Technologies Corporation. Other reagents were obtained from Xilong Reagent Company. Nanopure water (18.2 MΩ) was used in all experiments. All the chemicals were used as received without further purification.

### 2.2. Characterization

Transmission electron microscopy (TEM) images were obtained on a JEOL 3010 microscope. A small angle powder X-ray diffraction pattern of the MSN materials was obtained in a Scintag XDS-2000 powder diffractometer. N_2_ adsorption−desorption isotherm was obtained at 77 K on a Micromeritics ASAP 2010 sorptometer by static adsorption procedures. Zeta potential experiments were performed using a Malvern ZetaSizer Nano instrument. All fluorescence spectra were recorded on a Hitachi F-7000 FL spectrophotometer in PBS buffer. The confocal laser scanning microscopy (CLSM) images were obtained on a FluoView FV300, Olympus.

### 2.3. Synthesis of amino-functionalized mesoporous silica nanoparticle

MSN was synthesized by a base-catalysed sol–gel procedure [31]. Briefly, 1.00 g CTAB was first dissolved in 480 ml of deionized water and 3.5 ml NaOH solution (2.00 M) was added to the CTAB solution, after adjusting the solution temperature to 80°C. Then, 5.0 ml TEOS was added dropwise to the surfactant solution under vigorous stirring. After 60 min, 0.5 ml of APTES solution was slowly added to the mixture, and the reaction was processed for another 30 min. The product was subsequently washed, and redispersed with deionized water and ethanol several times. Surfactant templates were removed by calcination at 550°C for 5 h.

### 2.4. Doxorubicin loading and hyaluronic acid capping

In this work, DOX was used as a model guest drug to evaluate the loading and controlled releasing behaviour because of its fluorescence properties and water solubility. For the preparation of DOX-loaded MSN@HA, NH_2_-MSN (10 mg) was diffusion-filled with DOX by immersing the particles in a solution of DOX (500 µl, 0.01 M, pH 7.2) overnight, followed by centrifuging to remove the excess DOX solution. The residual particles were gently shaken with 4.0 ml 0.01 M MES solution (pH = 5.5) and added with 10 mg of HA. Then, 10 mg of EDC and 10 mg of NHS were added for amidation reaction, and the mixture was gently stirred for 4 h in an ambient temperature. The samples were centrifuged, rinsed by water and redispersed three times. The loading amount of DOX was calculated by subtracting the amount of DOX molecules remaining in the supernatant, and combined washings from the initial amount of DOX were added to the reaction.

### 2.5. Modification of mesoporous silica nanoparticles with gelatin and poly(ethylene glycol) polymers

The residual DOX-loaded MSN@HA particles were shaken with an aqueous gelatin solution (1 ml, 0.8%) at 50°C for 3 h. Then, deionized water (8 ml) at 4°C was poured into the mixture quickly. After two centrifugation/water rinsing/redispersion cycles, 50 µl of a 1% glutaraldehyde solution was added to cross-link the gelatin for 8 h. The samples were centrifuged, rinsed by water and redispersed three times. Then, 10 mg EDC and 10 mg NHS were dissolved in 500 µl of nanopure water and added to the nanoparticle mixture. After the reaction proceeded for 30 min, an mPEG amine solution (10 mg, 5 kDa) was added. The mixture was stirred at room temperature for 16 h, followed by centrifugation and washing with nanopure water. The sample was denoted as DOX/MSN@HA-gelatin-PEG (DOX/MHGP).

### 2.6. Doxorubicin release

For the *in vitro* release experiment, a small sample of DOX/MHGP was placed in a cuvette, which was then carefully filled with a 200 µl Hepes buffer (50 mM Hepes, 2 mM CaCl_2_, pH 7.4) that contained different enzymes. Subsequently, the release profiles of DOX molecules from the pores to aqueous solution were monitored via the fluorescence intensity of the DOX centred at 560 nm (*λ*_ex_ = 480 nm). Time-dependent release of DOX from the PGFMSN particles was studied at 37°C.

### 2.7. Confocal laser microscopic analysis

MDA-MB-231 cells and L02 cells at 4 × 10^5^ per well were seeded in cell culture dishes with glass bottom. The cells were seeded in DMEM media containing 10% (v/v) fetal bovine serum and cultured for 8 h. Then, half of the culture medium was removed, and different DOX-loaded nanoparticles were added in each well. The cells were co-incubated with DOX-loaded nanoparticles at 37°C for 3 h. The cells were observed using an Olympus confocal laser scanning microscope (FluoView FV1000). For each group, the same optical power intensity and image resolution were used.

### 2.8. Cytotoxicity test

MDA-MB-231 cells and L02 cells at 4 × 10^4^ per well were seeded in 96-well plates and cultured in DMEM media containing 10% (v/v) fetal bovine serum for 24 h (37°C, 5% CO_2_). Then, the culture medium was removed, and RPMI 1640 media (200 µl) containing a fixed amount of DOX-loaded nanoparticles were added in each well. The cells were co-incubated with DOX/MHGP at 37°C for 48 h. For comparison, the cell viability of unloaded MHGP, free DOX, DOX/MSN@gelatin-PEG was also investigated. After the incubation with particular nanoparticles, MTT solution (20 µl, 5 mg ml^−1^) was added to each well and the cells were further incubated for another 4 h. Then fluorescence readings were measured with an excitation wavelength of 560 nm and an emission wavelength of 590 nm using a microplate reader. Cells incubated in the absence of particles were used as the control. All the experiments were performed in triplicate for each group.

## 3. Results and discussion

**Figure 1.**
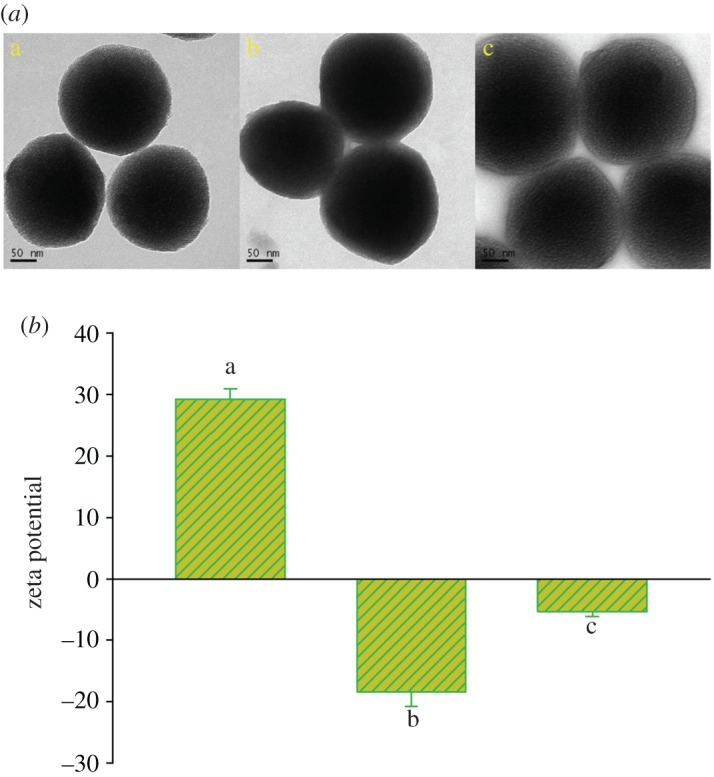
(*a*) The TEM and (*b*) zeta potential of (a) NH_2_-MSN, (b) MSN@HA and (c) MHGP.

According to the design, MSN was first synthesized by the sol–gel method, and the resulting particles were amino functionalized to form NH_2_-MSN. The powder X-ray diffraction (XRD) pattern shows four low-angle reflections typical of a hexagonal array which can be indexed as (100), (110) and (200) Bragg peaks (electronic supplementary material, figure S1). HA, working as a tumour-targeting ligand and pore blocker, was then introduced onto the surface of the nanoparticles to obtain the HA-functionalized MSN (denoted as MSN@HA) via the reaction between the amino groups of the NH_2_-MSN and the carboxyl group of HA. TEM images show that the resulting spherical NH_2_-MSN and MSN@HA exhibit a well-defined mesostructure (figure 1*a*). In the zeta potential measurements, NH_2_-MSN offered a positive zeta potential of 29.3 ± 1.7 mV. After HA was grafted, the surface charge was reversed to a negative value of −18.4 ± 2.4 mV (figure 1*b*). Meanwhile, the surface grafting of the nanoparticles was monitored by Fourier transform infrared (FTIR) spectra. As illustrated in electronic supplementary material, figure S2, MSN@HA shows that a strong absorption band at approximately 1556 cm^−1^ in the sample can be assigned to the stretching vibration of N−H bending of HA. However, there is no absorption band before the efficient immobilization of HA onto mesoporous silica, further indicating the successful coating process. Hereafter, the as-synthesized MSN@HA particles were capped with gelatin and modified with PEG. This procedure was also confirmed by electron microscopy image and zeta potential evidence. It is clear that the particle size was increased significantly. The value of zeta potential was changed to −5.4 ± 0.7 mV. Quantification of all modification processes was accomplished by the thermogravimetric analysis (TGA). As shown in electronic supplementary material, figure S3, the weight loss values of unloaded NH_2_-MSN, MSN@HA, MSN@HA@Gelatin and MHGP were 4.9%, 12.4%, 24.2% and 30.4%, respectively, when the temperature increased to 800°C. The immobilized mass percentage of HA, gelatin and PEG was approximately 7.5%, 11.8% and 6.2% respectively, which also reflected the successful modification of each functional unit.

**Figure 2.**
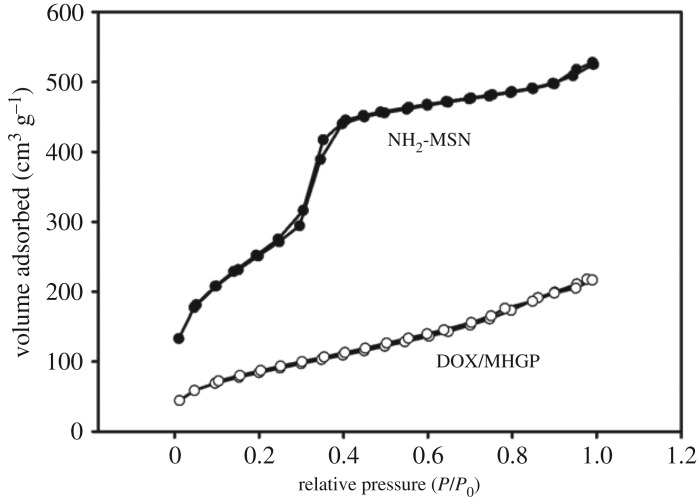
Nitrogen sorption isotherms of NH_2_-MSN and DOX/MHGP.

In order to investigate the bienzyme-responsive drug release of the MHGP system, DOX was then selected as a guest molecule to fabricate DOX/MHGP. The DOX loadings were proved by Brunauer−Emmett−Teller and Barrett−Joyner−Halenda analyses. As shown in figure 2, N_2_ adsorption−desorption isotherms of the NH_2_-MSN presented an adsorption step at an intermediate *P*/*P*_0_ value (0.2−0.4) typical of MCM-41-type structure. The surface area (284.9 m^2 ^g^−1^) and pore volume (0.37 cm^3 ^g^−1^) of DOX/MHGP show a sharp drop compared with the high surface area (936.6 m^2 ^g^−1^) and large pore volume (0.89 cm^3 ^g^−1^) for as-synthesized NH_2_-MSN. This result demonstrated that the pores of MHGP were filled with DOX. Based on the measurement of UV-vis absorption spectrum, the loading amount of DOX stored inside of MHGP was as high as 53.7 mmol g^−1^ SiO_2_.

**Figure 3.**
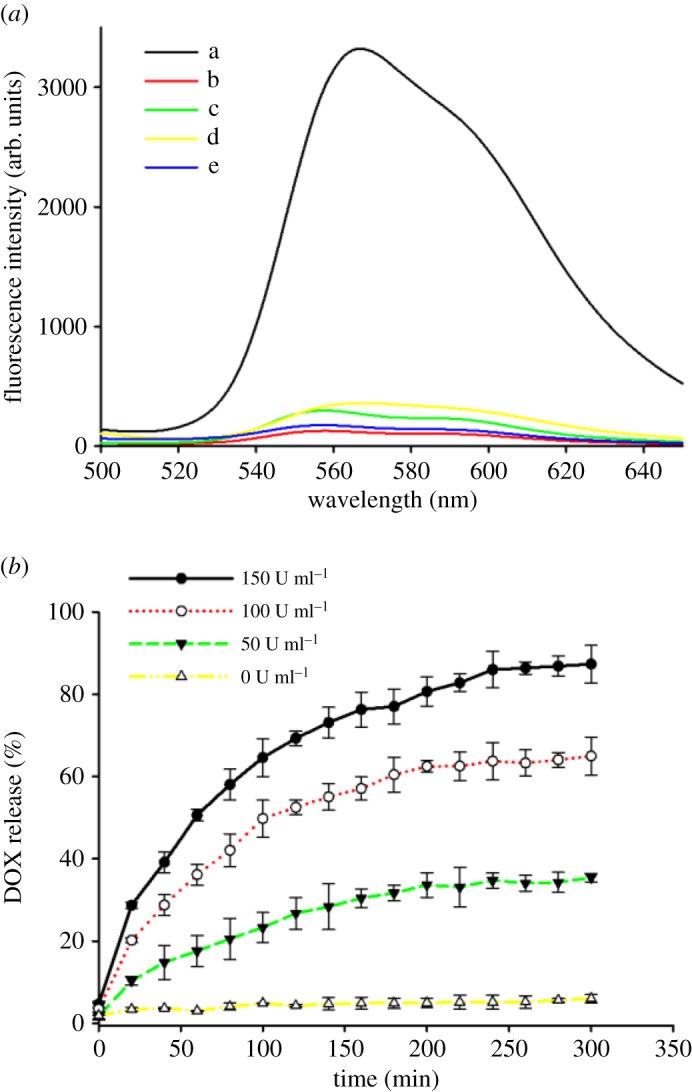
(*a*) Fluorescence emission spectra of released DOX from DOX/MHGP in PBS buffer with different conditions: a. DOX/MHGP treated with MMP-2 and Hyal-1; b. DOX/MHGP treated with MMP-2; c. DOX/MHGP treated with Hyal-1; d. DOX/MHGP treated with MMP-2 and Hyal-1 in the presence of MMP inhibitor; e. DOX/MHGP treated with MMP-2 and Hyal-1 in the presence of MMP inhibitor after Hyal-1 inactivation. (*b*) The kinetic profiles of the contents release in the presence of 10 µg ml^−1^ MMP-2 and different concentrations of Hyal-1.

The release behaviours of DOX were then tested by monitoring the fluorescence intensity at 560 nm (*λ*_ex_ = 480 nm). As shown in figure 3*a*, when both MMP-2 and Hyal-1 were introduced, a burst release of DOX from DOX/MHGP could be observed after 10 h from the strong fluorescence of supernatant-released DOX. However, negligible DOX release was obtained in the same amount of time without MMP-2 and Hyal-1. To further confirm that the release of DOX was attributed to the bienzymatic-mediated degradation, two additional experiments were carried out. In one experiment, MMP-2 in the presence of MMP inhibitor and Hyal-1 was added to DOX/MHGP for 10 h. In another experiment, the Hyal-1 was denatured by heating enzyme solutions at 90°C for 45 min before it was added to DOX/MHGP with MMP-2. As a result, only a small amount of DOX was released in the above experiments. As presented in figure 3*b*, the DOX/MHGP exhibited time- and Hyal-1-dependent release properties and approximately 6.0%, 35.4.1%, 65.9% and 87.3% DOX was released within 3 h at Hyal-1 concentration of 0, 50, 100, 150 U ml^−1^, respectively. These results confirmed that the bienzymatic-mediated degradation of HA and gelatin was the mechanism responsible for the opening of the mesopores.

**Figure 4.**
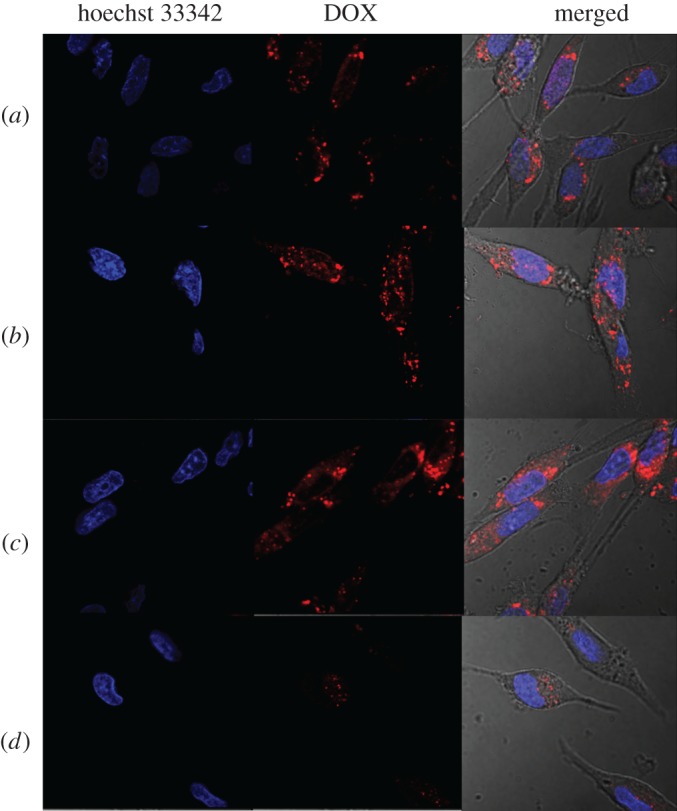
The confocal microscopy studies of the cellular uptake of different particles after incubation with L02 cells and MDA-MB-231 cells for 3 h at a concentration of 100 µg ml^−1^. (*a*) DOX/MHGP incubated with MDA-MB-231 cells in the presence of MMP inhibitor; (*b*) DOX/MSN@gelatin-PEG incubated with MDA-MB-231 cells; (*c*) DOX/MHGP incubated with MDA-MB-231 cells; (*d*) DOX/MHGP incubated with L02 cells. Red fluorescence arises from DOX and nuclei are stained with Hoechst 33342, showing blue fluorescence.

Targeted delivery to specific cells is essential for chemotherapy. In our system, HA served as a blocking and targeting agent. The cellular targeting efficiency was investigated by incubation with L02 (a hepatocyte line) and MDA-MB-231 (a human breast cancer cell line that CD44 over-expressed) cell line with DOX/MHGP. As can be seen in figure 4, a large amount of DOX/MHGP was internalized into the cells. By contrast, the fluorescence intensity distributed inside the cells is dramatically reduced when DOX/MHGP was incubated with MDA-MB-231 cells in the presence of MMP inhibitor, indicating that few particles were internalized by cells. This phenomenon was attributed to the detachment of PEG from DOX/MHGP and the HA-mediated endocytosis. MMP-2 overexpressed by MDA-MB-231 cells could hydrolyse the layer of gelatin, leading to the exposure of HA. The exposed HA on particles could significantly improve the specific targeting of the nanoparticle carrier towards CD44 receptors overexpressed MDA-MB-231 cells and facilitate the particle uptake. By contrast, MMP inhibitor inhibited the effect of MMP. So, the targeting HA was protected and the internalization of DOX/MHGP was significantly inhibited. In another control experiment, few nanoparticles were taken up into L02 cells because the expression level of MMP-2 is very low. In order to further verify the targeting role of HA, DOX/MSN@gelatin-PEG without HA coating was fabricated and incubated with MDA-MB-231 cells. As shown in figure 4, after 3 h of incubation, the red fluorescence of DOX was readily apparent within MDA-MB-231 cells. However, the red fluorescence of DOX/MSN@gelatin-PEG was weaker than that of DOX/MHGP, which clearly indicates that the DOX/MHGP had higher concentration within MDA-MB-231 cells than DOX/MSN@gelatin-PEG. Taken together, the HA-coated MSNs shows great advantage to target breast cancer cells and enhance cellular uptake.

**Figure 5.**
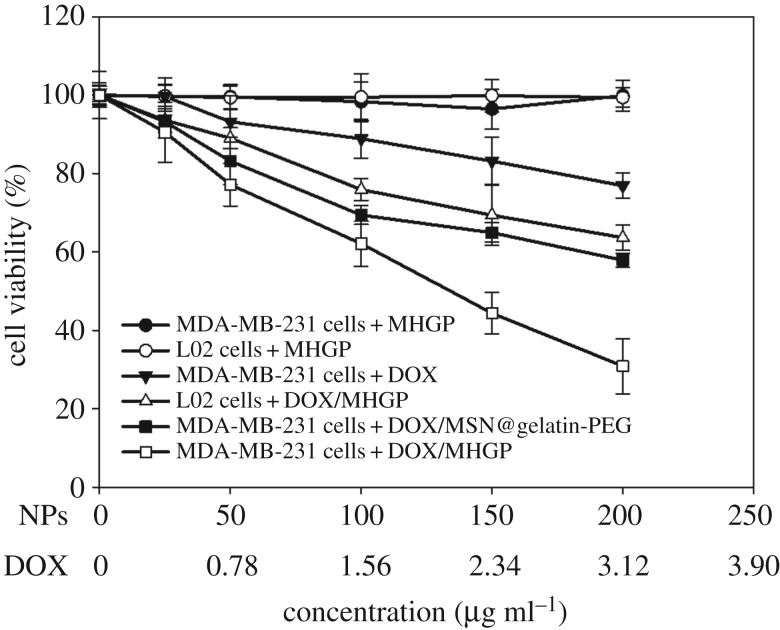
*In vitro* cytotoxicity assay curves for MDA-MB-231 cells and L02 cells obtained by plotting the cell viability percentage against the concentration of DOX.

To demonstrate anti-cancer effects of the bienzyme-responsive system, the MTT assay was used for quantitative testing of the cell viability. MDA-MB-231 cells were treated with free DOX, MHGP, DOX/MSN@gelatin-PEG or DOX/MHGP with the same concentration of DOX. Meanwhile, L02 cells were also treated with DOX/MHGP or MHGP as control groups. As shown in figure 5, almost no toxicity was observed when MDA-MB-231 cells and L02 cells were incubated with MHGP, indicating the excellent biocompatibility of MHGP. In the same concentration range, MDA-MB-231 cells treated with free DOX, DOX/MSN@gelatin-PEG and DOX/MHGP exhibited a DOX dosage-dependent cytotoxicity. However, DOX/MHGP showed higher cytotoxicity than free DOX and DOX/MSN@gelatin-PEG. In addition, much weaker cytotoxicity to L02 cells was also observed for DOX/MHGP than to MDA-MB-231 cells at the same concentration, which was the result of lack of CD44 receptors on the cell surface and a lower level of intracellular Hyal-1. The enhanced killing efficiency of DOX/MHGP indicated that the targeting of the nanoparticles into MDA-MB-231 cells through HA receptor endocytosis increases the internalization of nanoparticles and thus leads to a high percentage of DOX release in response to Hyal-1.

## 4. Conclusion

We have developed a unique DDS for bienzyme-responsive tumour targeting and drug-controlled release based on MMP-2-catalysed degradation of gelatin and Hyal-catalysed degradation of HA. Through the MEND strategy, this system can specifically target cancer cells responding to the up-regulated extracellular MMP-2 in tumours and provide enhanced cellular internalization via HA receptor-mediated endocytosis. Drug release was then triggered by Hyal-1 present in the tumour microenvironment. *In vitro* results indicated that this system achieved enhanced cellular uptake performance and remarkably killing efficiency to CD44-positive MDA-MB-231 cells. The excellent biocompatibility, cancer cell recognition ability and efficient intracellular drug release provide new horizons in clinical cancer therapy and cancer pharmaceutical development.

## Supplementary Material

Supporting Information

## Data accessibility

This article does not contain any additional data.
