# Supplementary material for: Mesoporous silica nanoparticle-based intelligent drug delivery system for bienzyme-responsive tumour targeting and controlled release
Source: R Soc Open Sci. 2018 Jan 10;5(1):170986. doi: 10.1098/rsos.170986 (PMC5792888; doi:10.1098/rsos.170986)
Supplement: Supporting Information [file rsos170986supp1.doc]

**Mesoporous Silica Nanoparticle-Based Intelligent Drug Delivery System for Bienzyme-Responsive Targeting to Cancer Cells and Controlled Release**

Yang Zhang and Juan Xu*

Department of Obstetrics and Gynecology, Tengzhou Central People's Hospital, No.181 Xingtan Road, Shandong 277599, P.R. China.

Email: xujuan2010tz@163.com


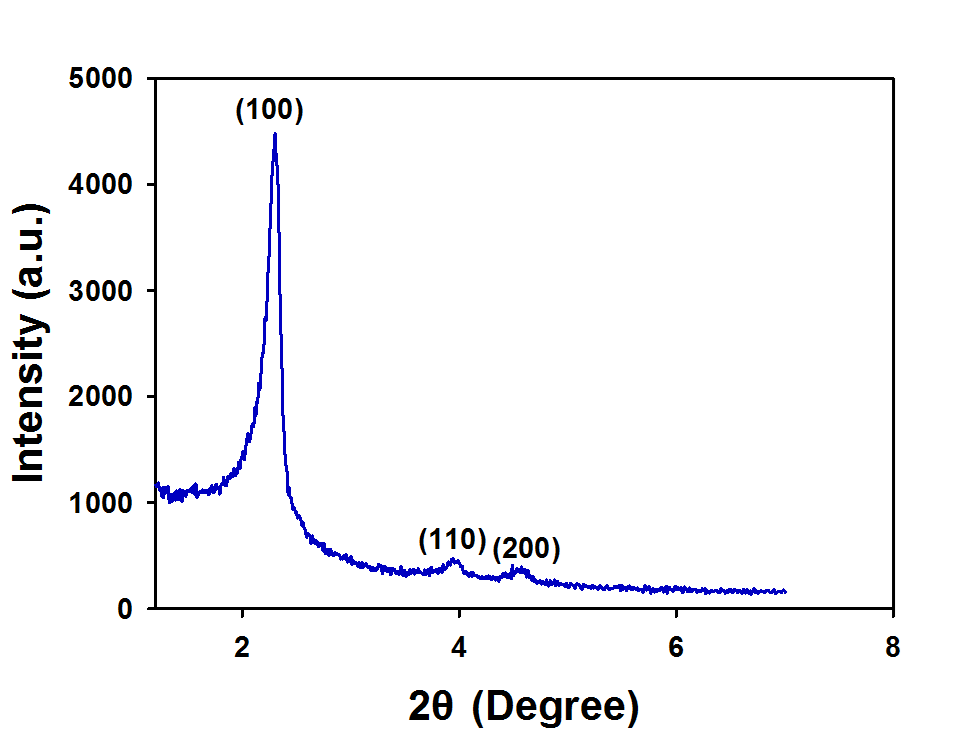


**Figure S1**. Powder X-ray pattern of NH_2_-MSN.


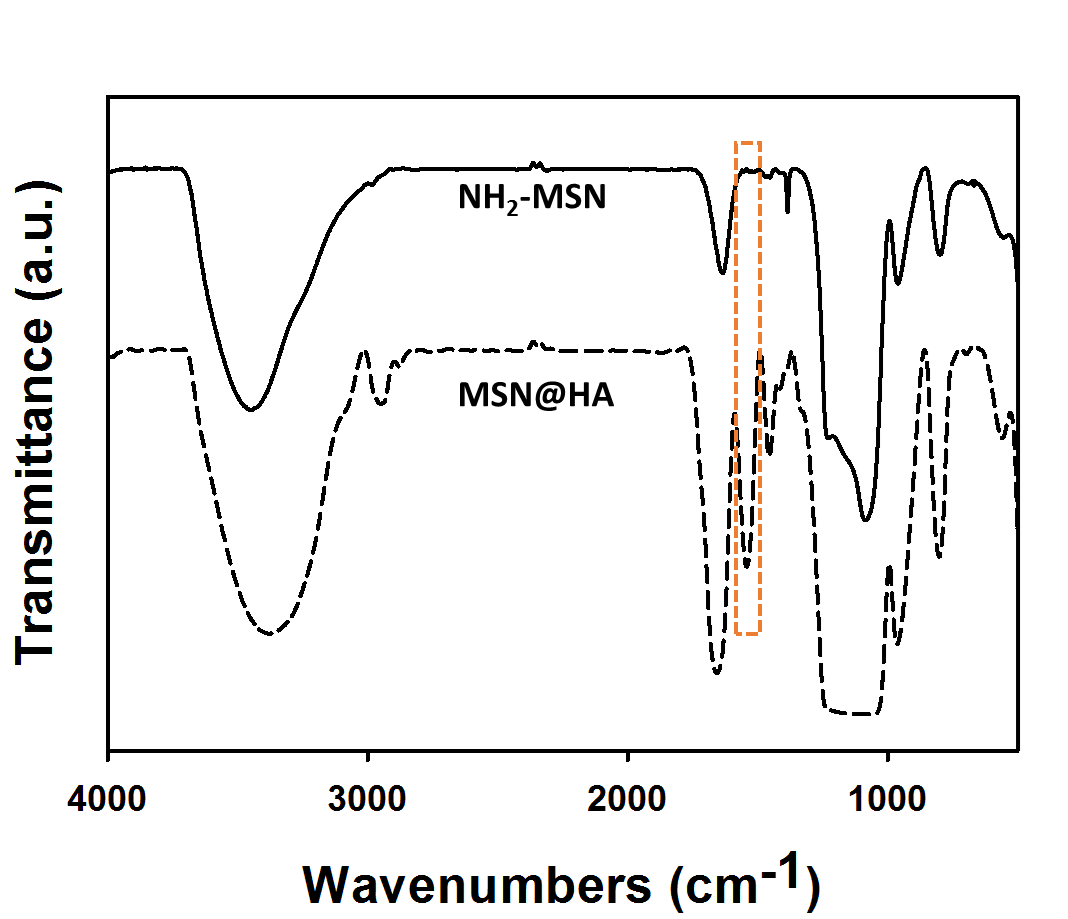


**Figure S2**. FTIR spectra of the samples NH_2_-MSN and MSN@HA.


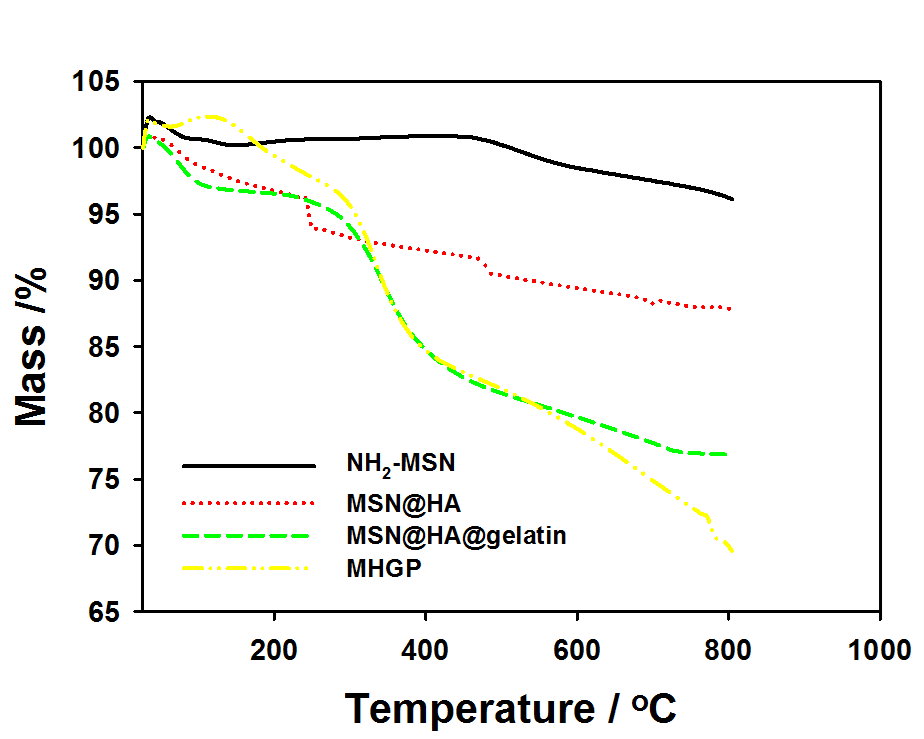


**Figure S3**. TGA curves of unloaded NH_2_-MSN, MSN@HA, MSN@HA@Gelatin, and MHGP
